# Early Tuberculosis Detection via Privacy-Preserving, Adaptive-Weighted Deep Models

**DOI:** 10.3390/diagnostics16020204

**Published:** 2026-01-08

**Authors:** Karim Gasmi, Afrah Alanazi, Najib Ben Aoun, Mohamed O. Altaieb, Alameen E. M. Abdalrahman, Omer Hamid, Sahar Almenwer, Lassaad Ben Ammar, Samia Yahyaoui, Manel Mrabet

**Affiliations:** 1 Department of Computer Science, College of Computer and Information Sciences, Jouf University, Sakaka 72388, Saudi Arabia; 2 Department of Information System, College of Computer and Information Sciences, Jouf University, Sakaka 72388, Saudi Arabia; 3 Faculty of Computing and Information, Al-Baha University, Alaqiq 65779-7738, Saudi Arabia; 4 Cybersecurity Department, College of Engineering and Information Technology, Buraydah Private Colleges, Buraydah 51418, Saudi Arabia; 5 College of Computer Engineering and Sciences, Prince Sattam bin Abdulaziz University, Al-Kharj 11942, Saudi Arabia; 6 Department of Physics, College of Science, Jouf University, Sakaka 72341, Aljouf, Saudi Arabia

**Keywords:** SDG 3, tuberculosis detection, ensemble learning, optimal algorithm

## Abstract

**Background:** Tuberculosis (TB) is a significant global health issue, particularly in resource-limited regions where radiological expertise is constrained. This project aims to develop a scalable deep learning system that safeguards privacy and achieves high accuracy in the early identification of tuberculosis using chest X-ray images. The objective is to implement federated learning with an adaptive-weighted ensemble optimised by a Genetic Algorithm (GA) to address the challenges of centralised training and single-model approaches. **Method:** We developed an ensemble learning method that combines multiple locally trained models to improve diagnostic consistency and reduce individual-model bias. An optimisation system that autonomously selected the optimal ensemble weights determined each model’s contribution to the final decision. A controlled augmentation process was employed to enhance the model’s robustness and reduce the likelihood of overfitting by introducing realistic alterations to appearance, geometry, and acquisition conditions. Federated learning facilitated collaboration among universities for training while ensuring data privacy was maintained during the establishment of the optimal ensemble at each location. In this system, just model parameters were transmitted, excluding patient photographs. This enabled the secure amalgamation of global data without revealing sensitive clinical information. Standard diagnostic metrics, including accuracy, sensitivity, precision, F1 score, AUC, and confusion matrices, were employed to evaluate the model’s performance. **Results:** The proposed federated, GA-optimized ensemble demonstrated superior performance compared with individual models and fixed-weight ensembles. The system achieved 98% accuracy, 97% F1 score, and 0.999 AUC, indicating highly reliable discrimination between TB-positive and typical cases. Federated learning preserved model robustness across heterogeneous data sources, while ensuring complete patient privacy. **Conclusions:** The proposed federated, GA-optimized ensemble achieves highly accurate and robust early tuberculosis detection while preserving patient privacy across distributed clinical sites. This scalable framework demonstrates strong potential for reliable AI-assisted TB screening in resource-limited healthcare settings.

## 1. Introduction

Tuberculosis (TB) remains one of the world’s deadliest infectious diseases, disproportionately affecting low- and middle-income countries where access to radiologists and advanced diagnostic facilities is limited. According to the WHO (https://www.who.int/news-room/fact-sheets/detail/tuberculosis, accessed on 1 August 2025) Global Tuberculosis Report 2024, nearly 10.3 million individuals developed TB and 1.4 million deaths occurred worldwide, with delayed detection contributing substantially to disease transmission and mortality [1,2]. Chest X-ray (CXR) imaging is a widely accessible and cost-effective screening tool, yet accurate interpretation requires specialized clinical expertise, making automated and scalable diagnostic support systems increasingly important.

Although conventional diagnostic procedures remain the primary approach to detecting tuberculosis, they have significant limitations. Sputum smear microscopy is readily available; however, it has low sensitivity, particularly in the early stages of tuberculosis (TB) or in extrapulmonary TB. Bacterial culture is considered the gold standard for diagnosis; however, the method takes several weeks to yield results, thereby delaying treatment initiation. GeneXpert MTB/RIF Ultra is a molecular assay characterized by faster turnaround times and improved sensitivity. However, these tests are expensive and challenging to implement in resource-limited settings [3]. Imaging modalities, including chest radiographs (CXR) and computed tomography (CT), are essential for patient screening and clinical assessment. The interpretation of these findings is subjective and depends on the radiologist’s expertise, and may vary [4,5]. The constraints above require developing novel diagnostic approaches that are precise, rapid, scalable, and readily accessible.

Deep learning (DL), particularly convolutional neural networks (CNNs), has revolutionized the domain by enabling the automatic extraction of information from unprocessed medical images [6]. Convolutional neural networks (CNNs), DenseNets, Inception architectures, and Vision Transformers (ViTs) have shown exceptional efficacy in tuberculosis (TB) classification tasks [7,8]. Transfer learning and fine-tuning on public datasets such as Shenzhen, Montgomery ( https://data.mendeley.com/datasets/8gf9vpkhgy/2, accessed on 1 August 2025), and TBX11K ( https://www.kaggle.com/datasets/surekhatammisetti/tbx11k, accessed on 1 August 2025) have improved diagnostic precision. In controlled environments, the accuracy of these approaches frequently equals or exceeds that of experienced radiologists. However, single-model frameworks remain susceptible to overfitting, domain shifts, and dataset imbalances.

However, most existing models rely on centralized training, where data must be collected from multiple institutions into a single repository. This approach raises critical concerns related to patient privacy, data governance regulations, and institutional data-sharing restrictions. Furthermore, centralized datasets often exhibit significant variability across imaging equipment, demographics, and labelling conventions, which can degrade real-world generalization. Another key challenge is that individual deep learning architectures capture different levels of visual abstractions, and no single model consistently performs best across domains. While ensemble learning can address this limitation, most studies rely on fixed or heuristic ensemble weights, neglecting the varying strengths of different architectures across datasets.

To address these challenges, we propose a federated, adaptive-weighted deep learning framework for early TB detection. The system integrates two architectures into a single ensemble whose weights are optimized using a multi-objective Genetic Algorithm (GA). Training occurs locally at participating hospitals, and only model updates—not raw images—are exchanged through the Federated Averaging (FedAvg) protocol. This enables privacy-preserving multi-centre collaboration while achieving strong diagnostic performance. The main contributions of this study are as follows:

- A privacy-preserving federated learning framework tailored for multi-institution TB detection without sharing patient data.
- A GA-optimized weighted ensemble that adaptively balances diverse deep models for improved diagnostic accuracy.
- A standardized, clinically motivated dataset preparation pipeline, including dat augmentation strategies.

The subsequent sections of this paper are organised as follows: Section 2 provides a comprehensive analysis of contemporary research in tuberculosis detection. In Section 3, we outline our proposed framework, including a detailed description of preprocessing procedures, data augmentation approaches, and an ensemble learning architecture that utilises deep classifiers and an optimisation algorithm. Section 4 outlines the experimental framework, including the dataset, evaluation metrics, and baseline configurations. Section 5 delineates the empirical findings. This involves comparing individual models with our ensemble and evaluating the effectiveness of the hybrid features. In summary, Section 6 summarises the key contributions and explores prospective directions for future research.

## 2. Related Work

Researchers have employed various computational intelligence methodologies over the years to address the challenges of recognizing and diagnosing tuberculosis (TB). There are two primary categories of these techniques: classical machine learning (ML) methods, which utilize handcrafted features and conventional classifiers, and deep learning (DL) methods, which autonomously create hierarchical representations from unprocessed data. The following sections provide additional details regarding the key contributions within each area.

### 2.1. Machine Learning Approaches for TB Diagnosis

Historically, computer-aided diagnosis of tuberculosis (TB) has been based on conventional machine learning (ML) techniques. The technologies in question often employ hand-made features extracted from medical imaging. These characteristics include shape, texture, and frequency domain descriptors. Once extracted, these features are assigned to classifiers such as support vector machines (SVMs), k-nearest neighbours (KNNs), Bayesian networks, or neural networks. Noise reduction, image enhancement, and segmentation are essential precursors to these processes, as the classifier’s feature quality depends on them. Machine learning algorithms often face limitations due to their reliance on domain-specific feature engineering and their inability to handle diverse datasets, even though they can be effective in some instances.

Hrizi et al. [9] developed an enhanced machine learning pipeline that emphasizes feature engineering, feature selection, and support vector machines (SVM). The authors assert that the concurrent optimization of the classifier’s hyperparameters and the feature subset improves accuracy. The researchers used support vector machines (SVMs) for final classification and genetic algorithms to identify discriminative wavelet-based texture features. The work conceptualizes tuberculosis (TB) diagnosis as a multiobjective optimization, striving to improve accuracy while reducing characteristics. The GA-tuned SVM outperformed numerous baselines, indicating that tuberculosis diagnosis requires meticulous feature selection and hyperparameter optimization, as demonstrated using ImageCLEF 2020 tuberculosis images.

Structured preprocessing, feature extraction, and the use of a statistical classifier are fundamental procedures in machine learning (ML), as demonstrated by the decision support system developed by Walia et al. [10] for tuberculosis (TB) diagnosis. Their objective was to facilitate an actionable diagnosis by capturing images. This indicated that machine learning was considered a beneficial instrument in clinical settings. The work illustrates pre-deep learning pipelines that primarily rely on task-specific postprocessing and curated features to ensure robustness against noise and staining variability.

Barros et al. [11] conducted a benchmarking study of multiple machine learning models for tuberculosis prediction rather than simple detection. They emphasized that the selection of the model and data partitioning methods can influence both discrimination and calibration. Their benchmarking methodology is effective for tuberculosis pipelines that need to link findings at the picture level to outcomes at the patient level. This benchmarking method emphasises the need for a uniform approach to assessing machine learning applications beyond screening, such as therapy planning and risk assessment.

Raof et al. [12] employed a clustering of K-means to differentiate Ziehl-Neelsen sputum smears, which facilitates the identification process of bacteria. This exemplifies segmentation and preprocessing, fundamental components of traditional machine learning operations. In contrast, Ayas et al. [13] introduced a bilevel thresholding method using a metaheuristic to address issues arising from staining variability and complex backgrounds. These techniques are characteristic of the preclassifier phase, during which contrast enhancement, denoising, and segmentation are employed to isolate bacilli or pulmonary structures before feature extraction and classification.

Priya and Srinivasan [14] investigated the problem of overlapping bacilli, a recurring phenomenon that reduces the efficacy of conventional machine learning (ML) methods. They presented a multilayer perceptron network capable of both image-level and object-level processing, accompanied by a specialized post-processing strategy designed to differentiate between adherent species. Although their models may lack complexity, the results they produce are highly significant. Accurate instance separation yields more precise measurements of shape and texture, thereby enhancing the performance of classifiers such as support vector machines (SVMs), K-nearest neighbours (KNNs), and probabilistic neural networks (PNNs).

Osman et al. [15] evaluated multiple iterations of the Extreme Learning Machine (ELM) to identify the most effective model for tuberculosis detection in tissue sections. ELM’s closed-form optimization and rapid training render it an excellent option for swift iterations on manually produced features, particularly in resource-constrained environments. Compared with conventional neural networks, ELMs are a robust benchmark for classical machine learning, particularly when rapid retraining and minimal hyperparameter adjustments are required in clinical deployment cycles.

Chithra and Jagatheeswari [16] introduced a Support Vector Neural Network optimized with a fractional crow search algorithm to categorize tuberculosis patients and assess the severity of their condition [16]. Incorporating a metaheuristic into the model-selection loop showed that hybrid feature selection and parameter optimization can significantly enhance the accuracy of classifiers compared to those provided by the program. This study facilitated the creation of optimization-aware machine learning architectures that prioritize search as a fundamental component of the pipeline, rather than a later adjustment phase.

Segmentation and feature-extraction methods inform traditional machine-learning classifiers. Numerous publications delineate these algorithms. The sections on review and methodology discuss microscopy thresholding techniques, including K-means, FCM, and rapid marching. Bayesian segmentation and metaheuristics represent more instances. The emphasis is placed on Fourier and wavelet qualities for texture representation in smear and chest images downstream. These possibilities are substantial, as tuberculosis manifestations can be difficult to detect and vary in size and severity. Robust frequency-domain descriptors enable traditional classifiers to maintain stability across variations in acquisition settings.

Standard Bayesian, SVM, PNN, and KNN models have been employed to filter segmented masks and identify genuine bacilli candidates post-segmentation. Reports indicate that overlapping bacilli diminish the capabilities of naïve learners unless instance-aware methods are used. These findings stimulated additional efforts to employ optimization approaches, such as genetic algorithms and crow search, alongside more organized feature sets to enhance separability and reduce the occurrence of false positives. The research team led by Arzhaeva et al. [17] used classification for computer-aided detection of tuberculosis (CAD-TB), based on global and local dissimilarity, in addition to microscopy. This classification predates contemporary concepts of metric learning. Their emphasis on dissimilarity across many scales indicates an effort to contextualize typical pipelines from the outset. Subsequently, deep learning techniques inherently adopt this notion, which remains beneficial for systems designed to incorporate these characteristics.

Ultimately, conventional pipelines underscore the importance of pretreatment in the rapid detection and stabilization of characteristics. Susanto et al. [18] argue that targeted pre-processing enhances the efficiency of lung-TB diagnosis, which is crucial for effective triage. Numerous studies have shown that image enhancement, segmentation, and feature extraction are the first standard procedures in machine learning-based tuberculosis computer-aided detection.

### 2.2. Deep Learning Approaches for TB Diagnosis

Deep learning (DL) has revolutionized tuberculosis (TB) diagnosis by enabling the direct extraction of features from raw chest radiographs and computed tomography (CT) images. Unlike conventional machine learning techniques, deep learning architectures such as convolutional neural networks (CNNs), DenseNets, and Vision Transformers (ViTs) can autonomously identify patterns at both low and high levels without requiring manual input creation. Transfer learning and ensemble methods have enhanced generalization by utilizing pre-trained models and integrating various architectures. These developments have established deep learning (DL) as the foremost paradigm for tuberculosis (TB) detection, providing improved accuracy, scalability, and adaptability across diverse imaging datasets.

Hwang et al. [19] were pioneers in demonstrating the efficacy of convolutional neural networks (CNNs) for tuberculosis (TB) detection on chest radiographs. Influenced by AlexNet, their convolutional neural network (CNN) autonomously acquires hierarchical features, eliminating the need for manual engineering. It outperformed conventional classifiers, achieving elevated sensitivity and specificity. This groundbreaking study demonstrated that end-to-end learning pipelines can identify subtle indicators of illness that manually crafted features may have missed, thereby promoting the widespread adoption of deep learning in tuberculosis detection.

Lakhani and Sundaram expanded on this research by training multiple convolutional neural networks (CNNs) and integrating them into an ensemble to detect pulmonary tuberculosis (TB) using chest radiographs [20]. Evaluations on the Montgomery and Shenzhen datasets showed that the ensemble achieved an AUC of 0.99, comparable to the accuracy of experienced radiologists. This study demonstrates the potential of deep learning and highlights the effectiveness of ensemble techniques in overcoming the limitations of individual convolutional neural networks (CNNs). This work thus foreshadowed the current fascination with hybrid designs.

Gupta et al. [21] proposed a CNN architecture for diagnosing tuberculosis (TB) from chest radiographs. This architecture achieved an accuracy of 99.05% in the benchmark datasets. Their results demonstrated that a meticulously constructed convolutional neural network (CNN) can perform effectively when its hyperparameters are properly configured. In contrast, their study highlighted the vulnerability of individual models to dataset bias, prompting a subsequent transition towards ensembles and transfer learning to attain generalization across domains.

Nguyen et al. [22] proposed a transfer learning approach that entailed fine-tuning pre-trained deep learning models, such as VGGNet and ResNet, for tuberculosis classification. Leveraging the extensive, pre-trained weights from ImageNet, their models rapidly adjusted to the constrained terabytes of data while maintaining considerable accuracy. This study revealed that transfer learning significantly reduces training time and mitigates the challenges associated with limited data, thereby making deep learning viable for smaller tuberculosis-related datasets.

Eisentraut et al. [23] introduced a novel deep learning pipeline that is built on preprocessing. This pipeline employs a Gaussian filter on chest X-ray images as a “software lens” before feeding them into ResNet-50. The application of the Gaussian filter enhanced the visibility of the lung region and reduced background noise, thereby significantly improving the categorization efficacy. Their methodology achieved a 99.2% accuracy in a 5-fold cross-validation, exceeding the accuracy of previous studies employing convolutional neural networks. This study demonstrates that applying appropriate preprocessing techniques can improve deep learning models’ ability to distinguish between entities. The findings indicated that incorporating lightweight preprocessing filters into convolutional neural networks (CNNs) may be more effective than employing deeper networks.

Jaeger et al. [24] conducted a study examining the integration of convolutional neural networks (CNNs) with lung segmentation for identification of tuberculosis (TB). By segmenting lung fields into smaller regions before inputting the images into convolutional neural networks (CNNs), they reduced unnecessary features and improved classification accuracy. Their research demonstrates that hybrid segmentation-convolutional neural network (CNN) architectures effectively reduce noise and improve the interpretability of deep learning (DL) pipelines over time.

Steiner et al. [25] investigated the use of deep learning (DL) for the identification of anomalies by employing DLAD (Deep Learning for Abnormality identification) models on chest radiographs. Their findings demonstrated that general-purpose deep learning screening approaches can be adapted for pneumothorax applications with minimal retraining, although research has not been exclusively focused on pneumothorax. This tendency indicates frameworks capable of identifying numerous diseases, including tuberculosis, which is merely one of several targets. This facilitates practical application in everyday life.

Nijiati and colleagues [26] utilized longitudinal CT scans, combined with convolutional neural networks (CNNs) and radiomic characteristics, to predict tuberculosis treatment outcomes. This study extends from diagnosis to include prognosis. Their model effectively distinguished between individuals predicted to have poor therapeutic responses and those expected to exhibit favorable responses, with an AUC of approximately 0.93. This study is essential because it extends the application of deep learning from basic anomaly detection to clinical outcome prediction, thereby transforming artificial intelligence into both a screening tool and an aid for clinical decision-making in treatment.

### 2.3. Summary of Related Studies

Table 1 presents a concise comparison of previous investigations. This provides a concise summary of the prevalent studies that employ machine learning and deep learning techniques to detect tuberculosis. The table illustrates the various approaches, datasets, and performance indicators. It provides a brief comparison between traditional machine learning models that use manually engineered features and contemporary deep learning architectures that learn features automatically. It also demonstrates an inclination toward hybrid and ensemble-based solutions that consistently outperform a single model. This illustrates the intended function of the proposed deep learning architecture within the ensemble.

### 2.4. Ensemble Learning Approaches for TB Diagnosis

Ensemble learning has demonstrated efficacy in detecting tuberculosis (TB) by leveraging the complementary strengths of multiple classifiers to improve accuracy and robustness. Ensemble approaches employ several learners, either traditional machine learning classifiers or deep learning architectures, to reduce variance, mitigate overfitting, and improve generalization across diverse datasets. This differs from reliance on a single model. The expanding body of evidence supports the notion that ensembles consistently outperform individual models. This is especially relevant for chest radiographs or CT scans, where disease patterns are difficult to discern and heterogeneous.

Hooda et al. were among the first research teams to validate the use of ensembles for tuberculosis diagnosis. They achieved this by integrating AlexNet, GoogleNet, and ResNet into a cohesive framework [7]. Their ensemble surpassed individual convolutional neural networks (CNNs), illustrating that model fusion can more efficiently capture both shallow and deep feature hierarchies. Lakhani and Sundaram [20] developed an ensemble of multiple convolutional neural networks (CNNs) in a similar manner. The Montgomery and Shenzhen datasets were utilised to train these CNNs. Their methodology achieved an AUC of 0.99, comparable to that of professional radiologists. This demonstrated that ensemble averaging can eliminate model bias, enhancing its reliability for diagnostic purposes.

Ben Ammar, Gasmi, and Ben Ltaifa [8] developed a hybrid ensemble that integrates EfficientNet and Vision Transformers (ViTs) for tuberculosis (TB) detection. This marked the first application of ensemble learning to contemporary structures. Their investigation of public datasets demonstrated that employing both convolutional neural networks (CNNs) for local details and transformers for global context significantly improved network accuracy compared with a single network type. The findings of this study indicate that ensembles can incorporate various designs, rather than solely different iterations of the same model family.

Investigations in the extensive field of medical imaging further validate these results. Gupta et al. [21] achieved notable accuracy with individual CNNs; however, recent studies indicate that combining multiple CNNs of this type improves resilience, particularly on multicenter datasets. Eisentraut et al. [23] provided indirect evidence that supports the effectiveness of ensemble-style thinking. They demonstrated that ResNet50, when combined with Gaussian preprocessing, achieved state-of-the-art performance. This conclusion suggests that the results could be improved by employing a range of preprocessing strategies in ensembles. Furthermore, ensemble approaches are widely used for classifying various lung disorders [28,29], demonstrating their scalability across multiple disease scenarios.

In general, the use of ensemble learning significantly advances the transition from research-grade models to clinically applicable diagnostic tools, as presented in the Table 2. Ensembles can transcend the limitations of individual learners and establish a foundation for more robust frameworks by integrating the predictive capabilities of several models. This is particularly applicable to ensembles that utilize privacy-preserving approaches, such as federated learning.

Despite notable advancements in deep learning, ensemble methodologies, and machine learning, numerous concerns remain unresolved. Conventional machine learning methods rely heavily on hand-crafted features and do not apply to various imaging scenarios. In contrast, individual deep learning architectures often encounter issues related to overfitting and dataset bias. Most current ensembles employ basic aggregation techniques, such as majority voting or averaging, to consolidate multiple models, thereby enhancing their stability and robustness. However, many existing ensembles do not account for how each model can optimally contribute. While numerous studies have explored CNN-based architectures, hybrid CNN-transformer models, or ensemble strategies for tuberculosis detection, these approaches are predominantly conducted under centralized training paradigms. As a result, they do not address the real-world constraints associated with hospital-level data silos, legal restrictions on patient information, or domain shifts across datasets from different imaging equipment and demographic populations. Moreover, existing ensembles typically use uniform or heuristic weights that do not account for model-specific strengths or calibration differences. Our method distinguishes itself by presenting the first federated, GA-optimized ensemble for TB detection, enabling adaptive combination of diverse deep architectures while ensuring that training updates remain local to each institution. This fills an important gap in the literature by offering a practical, privacy-preserving, and performance-enhanced framework suitable for deployment in cross-institutional clinical collaborations.

## 3. Proposed Approach for Tuberculosis Classification

This paper introduces a federated ensemble deep learning system that combines sophisticated topologies, optimization-driven weighting, and privacy-preserving training for the diagnosis of tuberculosis (TB) using chest radiography (CXR) and computed tomography (CT) imagery. Our strategy primarily leverages the synergistic advantages of multiple deep learning models, each tailored to specific feature representations, while mitigating their limitations through appropriate ensemble weighting and federated learning. Initially, we describe the dataset, detailing its composition and clinical diversity. We employ a sophisticated data augmentation process to enhance our models’ robustness. This pipeline intentionally increases variability and addresses class imbalance. Subsequently, we provide an in-depth analysis of the deep learning models used, focusing on convolutional neural networks (CNNs), EfficientNet, and ResNet-50. The models are trained individually and then combined in pairs using ensemble techniques that leverage complementary patterns. We employ a genetic approach to enhance the weight allocation of each base learner, thereby optimizing the ensemble output. This ensures that the ensemble achieves the most precise and generalizable predictions. Ultimately, we employ federated learning to preserve data confidentiality and enable collaboration among multiple institutions for tuberculosis detection, without consolidating sensitive patient records in a single repository. This multi-tiered approach ensures precision, resilience, and clinical applicability across healthcare settings with varying resource availability.

**Figure 1:**
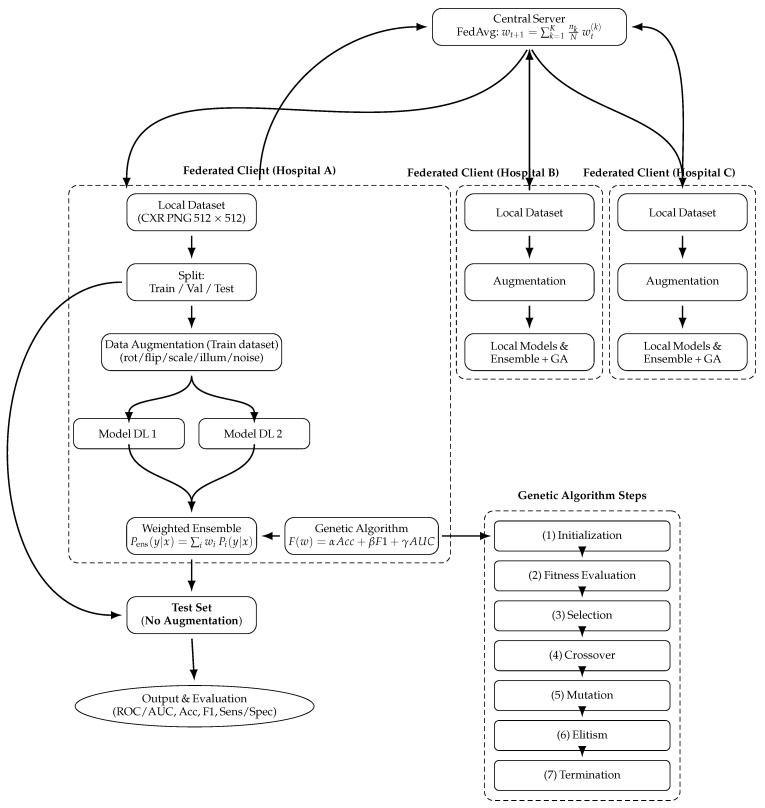
Proposed federated ensemble framework for tuberculosis classification (GA-optimized weights and FedAvg). Data augmentation is applied only during training; the test set remains unaugmented and is used exclusively for final evaluation.

Figure 1 illustrates the complete federated ensemble architecture used in this study. The process begins at each participating hospital, where the local dataset undergoes standardized preprocessing and augmentation. Each client trains multiple deep learning models. The softmax outputs of these models are combined via a weighted ensemble, with the optimal weights determined by a genetic algorithm. The optimized ensemble predictions are then transmitted to the central server, where the Federated Averaging (FedAvg) algorithm aggregates the local model updates into a global model. This process ensures collaborative learning across institutions while maintaining strict data privacy, as no raw patient images ever leave the local clients.

### 3.1. Dataset Description

We used the Tuberculosis (TB) Chest X-ray Database in our study [24,30,31,32,33]. Hamad Medical Corporation, Bangladeshi doctors, Qatar University, the University of Dhaka, and others from Malaysia contributed to the development of this database. This dataset is a highly reliable resource for tuberculosis (TB) research, containing 4200 chest X-ray (CXR) images. The dataset consists of 700 tuberculosis-positive patients and 3500 healthy controls (https://www.kaggle.com/datasets/tawsifurrahman/tuberculosis-tb-chest-xray-dataset, accessed on 1 August 2025).

**Figure 2:**
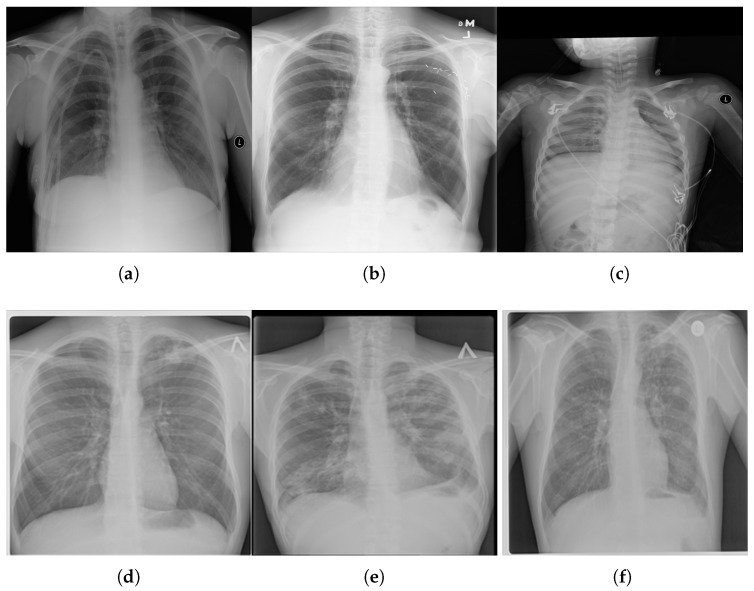
Examples of chest X-ray images: normal (**top row**) and tuberculosis (**bottom row**). (**a**) Normal 1; (**b**) Normal 2; (**c**) Normal 3; (**d**) Tuberculosis 1; (**e**) Tuberculosis 2; (**f**) Tuberculosis 3.

The varied sources make it more reliable for training and generalizing AI models. This makes it an excellent choice for creating federated deep learning methods. Some examples are presented in Figure 2.

### 3.2. Data Augmentation

**Figure 3:**
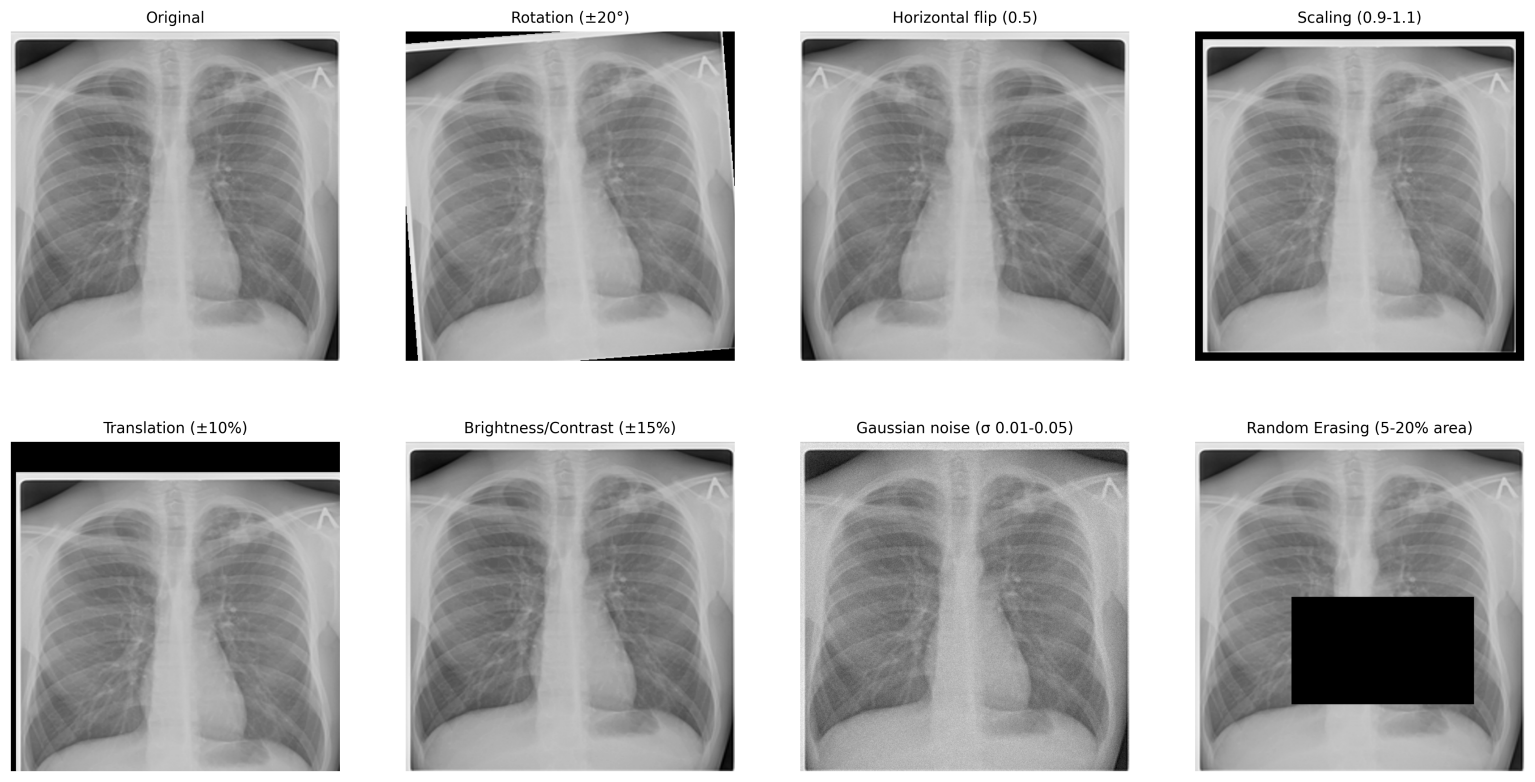
Augmentation Samples.

Deep learning algorithms often struggle when trained on small, imbalanced datasets. To mitigate overfitting and enhance the model’s generalizability, we employed a comprehensive data augmentation technique. The augmentation technique included random rotations, horizontal flips, scaling, and translations to simulate the unpredictability introduced by patient positioning variability. We employed random cropping, Gaussian noise, and brightness and contrast adjustments to simulate the variations inherent in various imaging instruments and acquisition techniques. Figure 3 presents representative examples of transformed images generated during the augmentation pipeline, including random rotation, flipping, scaling, brightness and contrast adjustments, Gaussian noise addition, and random occlusions. These transformations simulate realistic radiographic variability encountered in clinical imaging, thereby improving model robustness.

To replicate genuine radiographic inconsistencies, such as overlaying tissue or motion artifacts, simulated occlusions, and localized blurring were applied to the radiographic images to identify abnormalities indicative of tuberculosis. The enhancements were dynamically applied throughout training, ensuring that each epoch introduced distinct modifications to the images. This strategy enhanced the diversity of the training set, ensured equal representation of the regular and TB-positive groups, and mitigated bias against over-represented samples. Incorporating these augmentations enabled our models to handle previously unencountered clinical scenarios, thereby enhancing their utility in real-world diagnostic settings. To ensure a fair evaluation protocol, each hospital client performs a stratified split of its dataset into 70% training, 10% validation, and 20% testing. Data augmentation is applied exclusively to the training set to prevent artificial performance gains. Validation and test sets remain completely untouched by augmentation operations, ensuring unbiased evaluation.

### 3.3. Deep Learning Models

We implement seven distinct deep learning architectures to jointly identify local and global radiographic patterns associated with tuberculosis on chest radiographs. The models are initialized using weights from ImageNet and subsequently improved on the TB Chest X-ray Database previously discussed. This ensures effective convergence on the constrained medical data. We define the following terms: an input image is denoted as $x\in R^{H\times W\times C}$, a convolution kernel is represented by *K*, a channelwise nonlinearity is expressed as σ(·) (ReLU or its derivatives), batch normalization is indicated as BN(·), and max pooling is denoted as MP(·). Model-specific equations define the primary computational component of each network. This chapter employs the identical dataset references as the preceding sections to ensure robust repeatability and context.

Convolutional Neural Networks (CNNs):

Fundamental to standard Convolutional Neural Networks (CNNs) is the use of layered convolutions and pooling to systematically identify lung patterns, such as nodules, cavities, and consolidations of low to moderate severity. A convolutional layer with a single bias *b* is

(1) $$F_{i,j}^{\left(l\right)}=\sigma \;\left(\sum_{u=-\lfloor k/2\rfloor }^{\left\lfloor k/2\right\rfloor }\sum_{v=-\lfloor k/2\rfloor }^{\left\lfloor k/2\right\rfloor }K_{u,v}^{\left(l\right)}\cdot F_{i+u,\;j+v}^{\left(l-1\right)}+b^{\left(l\right)}\right),$$

followed by spatial downsampling

(2) $$F_{i,j}^{\left(l+1\right)}={\mathrm{MP}}_{p\times p}\;{\left(F^{\left(l\right)}\right)}_{i,j},$$

and a final softmax head for *C* classes:

(3) $$P\left(y=c|x\right)=\frac{\exp(z_{c})}{\sum_{c^{\prime }=1}^{C}\exp\left(z_{c^{\prime }}\right)}.$$

These CNNs provide strong baselines for subsequent ensembles and federated training, grounded in the dataset described in [24,30,31,32,33].

ResNet50 [34]:

ResNet introduces identity shortcut connections to ease optimization of very deep nets. A residual block with transformation F(·;Θ) is

(4) $$y=F\left(x;\Theta \right)+x,\;F\left(x;\Theta \right)=W_{3}\;\sigma \;\left(\mathrm{BN}\;\left(W_{2}\;\sigma \;\left(\mathrm{BN}(W_{1}x)\right)\right)\right),$$

where $\left(W_{1},W_{2},W_{3}\right)$ form the bottleneck (1×1–3×3–1×1) convolutions. The additive identity path stabilizes gradients and improves global context capture over the TB dataset splits [24,30,31,32,33].

EfficientNet [35]:

EfficientNet scales depth (*d*), width (*w*), and resolution (*r*) with a single compound coefficient ϕ:

(5) $$d=\alpha^{\phi },\;w=\beta^{\phi },\;r=\gamma^{\phi },\;s.t.\;\alpha \beta^{2}\gamma^{2}\approx 2,\;\alpha ,\beta ,\gamma >1,$$

and leverages MBConv with squeeze-and-excitation (SE). For an intermediate tensor Z, SE computes

(6) $$s=\sigma \;\left(W_{2}\;\delta \;\left(W_{1}\;\mathrm{GAP}\left(Z\right)\right)\right),\;\tilde{Z}=s⊙Z,$$

where GAP is global average pooling, δ is ReLU, and ⊙ is channel-wise scaling. This compound scaling offers high accuracy–efficiency trade-offs for real-world deployments on the same data [24,30,31,32,33].

InceptionV3 [36]:

Inception factorizes convolutions and aggregates multiscale branches. Given parallel branches ${\left\{B_{m}\left(\cdot \right)\right\}}_{m=1}^{M}$ (e.g., 1×1, 3×3, factorized 1×n + n×1, and pooled projections), the module output concatenates features along channels:

(7) $$Y=\mathrm{Concat}\left(B_{1}\left(X\right),\;B_{2}\left(X\right),\;\ldots ,\;B_{M}\left(X\right)\right).$$

Factorization reduces computation while preserving the discriminative capacity for various scales of TB lesions on the referenced data set [24,30,31,32,33].

Xception [37]:

Xception employs separable convolutions in depth, rather than conventional convolutions. These involve point-wise mixing after depth-wise spatial filtering.

(8) $$U_{i,j,c}=\sum_{u,v}K_{u,v}^{\left(c\right)}\cdot X_{i+u,\;j+v,\;c},\;Y_{i,j,c^{\prime }}=\sum_{c}W_{c}^{\left(c^{\prime }\right)}\cdot U_{i,j,c}.$$

Residual connections are frequently located adjacent to stacks of separable convolutions. This concept is advantageous for our TB splits, as it enhances parameter functionality while preserving the channel’s semantic integrity.

MobileNetV3-Large [38]:

MobileNetV3 couples inverted residual blocks, depthwise separable convolutions, SE, and the hard-swish activation:

(9) $$h\text{-}\mathrm{swish}\left(x\right)=x\cdot \frac{\mathrm{ReLU}6(x+3)}{6}.$$

An inverted residual block (stride *s*) can be summarized as

(10) $$Y=\underset{\mathrm{expand}}{\underbrace{{\mathrm{PW}}_{↑}\;\circ \;\mathrm{BN}\;\circ \;\delta }}\;\circ \;\underset{\mathrm{depthwise}\;+\;\mathrm{SE}}{\underbrace{{\mathrm{DW}}_{k\times k,s}\;\circ \;\mathrm{BN}\;\circ \;\mathrm{SE}}}\;\circ \;\underset{\mathrm{project}}{\underbrace{{\mathrm{PW}}_{↓}\;\circ \;\mathrm{BN}}}\;\left(X\right),$$

with a residual connection added when the shapes match. This yields strong trade-offs between accuracy and latency for potential point-of-care TB screening on the same dataset.

VGG16 [39]:

VGG16 uses uniform 3×3 convolutions stacked before pooling. A generic stage with *m* conv layers is

(11) $$F^{\left(t+q\right)}=\sigma \;\left(\mathrm{BN}(W^{\left(t+q\right)}*F^{\left(t+q-1\right)}+b^{\left(t+q\right)})\right),\;q=1,\ldots ,m,\;F^{\left(t+m+1\right)}={\mathrm{MP}}_{2\times 2}\left(F^{\left(t+m\right)}\right),$$

and the spatial dimension after a conv layer follows.

(12) $$H_{\mathrm{out}}=\left\lfloor \frac{H_{\mathrm{in}}+2p-k}{s}\right\rfloor +1,\;W_{\mathrm{out}}=\left\lfloor \frac{W_{\mathrm{in}}+2p-k}{s}\right\rfloor +1.$$

The regular and deep stack provide a robust baseline whose features combine well in our ensembles for the dataset in [24,30,31,32,33].

This collection of models offers numerous compatible inductive biases, including VGG and CNN for local texture, ResNet for deep global hierarchies, EfficientNet for balanced scaling, Inception/Xception for multiscale and separable filtering, and MobileNetV3 for efficient deployment. The efficacy of the weighted ensembles and GA-optimized fusion in our federated pipeline depends on their degree of differentiation.

### 3.4. Ensemble Learning

We employed an ensemble technique that combines predictions from design pairings to leverage each model’s strengths. A weighted averaging method is used to integrate the softmax outputs of two distinct models to form an ensemble.

(13) $$P_{ensemble}\left(y|x\right)=\sum_{i=1}^{n}w_{i}P_{i}\left(y|x\right),\;\sum_{i=1}^{n}w_{i}=1,$$

where $w_{i}$ is the weight assigned to the *i*-th model and $P_{i}\left(y|x\right)$ represents the probability that the *i*-th model will generate a forecast. This technique enables more robust models to exert a greater influence on the final decision than conventional majority voting.

By methodically integrating models, our approach consistently surpasses baselines that utilize a singular architecture. This results in improved generalization across various patient populations.

### 3.5. Genetic Algorithm for Optimal Weight Selection

A genetic Algorithm (GA) was used to determine the optimal method to assign weights to the ensemble. A chromosome represents each potential solution, while genes denote the weight values assigned to the fundamental models.

The genetic algorithm uses selection, crossover, and mutation to derive solutions. The fitness function evaluates the adequacy of each weight vector to meet the requirements:

(14) F(w)=α·Acc+β·F1+γ·AUC,

where Acc denotes accuracy, F1 signifies the F1 score, and AUC represents the area under the ROC curve. The parameters α, β, and γ determine the relative significance of each statistic relative to the others.

This optimization strategy prevents local minima and ensures that the ensemble weights accurately reflect the contribution of each model, thereby enhancing classification robustness.

**Genetic Algorithm (GA) Steps:**

1. **Initialization.** Create an initial population $P^{\left(0\right)}={\left\{w^{\left(j\right)}\right\}}_{j=1}^{M}$ of weight vectors on the simplex $\Delta =\{\;w\in R^{n}|w_{i}\geq 0,\;\sum_{i=1}^{n}w_{i}=1\;\}$ (e.g., Dirichlet sampling or non-negative random vectors followed by $w\leftarrow w/\sum_{i}w_{i}$).
2. **Fitness Evaluation.** For each $w^{\left(j\right)}$, compute the validation performance of the weighted ensemble
  $$P_{\mathrm{ens}}\left(y|x\right)=\sum_{i=1}^{n}w_{i}\;P_{i}\left(y|x\right),$$
  And score it with multi-objective fitness.
  F(w)=αAcc+βF1+γAUC,
  with α,β,γ≥0 (optionally α+β+γ=1).
3. **Selection.** Employ tournament selection or roulette wheel selection to select parents from $P^{\left(t\right)}$. Ensure that elevated F(w) is prioritized while maintaining engagement (selection pressure through tournament size or rank-based probability).
4. **Crossover.** Produce offspring by convex recombination of two parents $w^{\left(p1\right)}$ and $w^{\left(p2\right)}$:
  $$\tilde{w}=\lambda \;w^{\left(p1\right)}+\left(1-\lambda \right)\;w^{\left(p2\right)},\;\lambda ∼U\left(0,1\right),$$
  then renormalize $\tilde{w}\leftarrow \tilde{w}/\sum_{i}{\tilde{w}}_{i}$ to keep $\tilde{w}\in \Delta$.
5. **Mutation.** Perturb genes with probability $p_{m}$ (e.g., Gaussian noise $\epsilon_{i}\;∼\;N\left(0,\sigma^{2}\right)$ or Dirichlet smoothing):
  $$w_{i}\leftarrow \max\left(0,\;w_{i}+\epsilon_{i}\right),\;w\leftarrow \frac{w}{\sum_{i}w_{i}},$$
  To maintain nonnegativity and the simplex constraint.
6. **Elitism.** Copy the top-*E* fittest individuals from $P^{\left(t\right)}$ directly into $P^{\left(t+1\right)}$ to prevent loss of high-quality solutions.
7. **Termination (and Output).** The method must cease if the variation in F(w) across *G* generations is below a specified tolerance level, τ, or if *t* is greater than or equal to *T*. Retrieve the optimal $w^{⋆}$ and incorporate it into the ensemble.

$$P_{\mathrm{ens}}\left(y|x\right)=\sum_{i=1}^{n}w_{i}^{⋆}\;P_{i}\left(y|x\right).$$

Typical hyperparameters: The mutation scale is indicated by σ; elitism by *E*; population size by *M*; maximum generations by *T*; crossover probability by $p_{c}$; mutation probability by $p_{m}$; and patience by *G*. This genetic algorithm reliably explores the limited weight space, and the ensembles it generates are robust across the many TB sources used in this study.

The choice of a genetic algorithm (GA) for ensemble weight optimization is motivated by the inherent properties of the weight search space. Ensemble weight vectors lie on a constrained simplex and often exhibit non-linear interactions between model outputs. This landscape is typically multimodal, meaning that gradient-based optimization strategies can easily converge to suboptimal local minima. Furthermore, ensemble weights do not benefit from backpropagation because they are not trainable parameters. GAs, in contrast, perform population-based stochastic search, enabling efficient exploration of complex, discontinuous spaces without requiring differentiability. They naturally support multi-objective optimization, allowing our fitness function to simultaneously maximize accuracy, F1 score, and AUC. This makes GAs particularly well suited to federated environments, where the optimal model combination may differ across institutions due to dataset heterogeneity.

### 3.6. Federated Learning

We included Federated Learning (FL) into our system to enhance data privacy and facilitate scalability. In conventional centralized learning, the aggregation of patient data poses legal compliance and patient privacy concerns. FL addresses this issue by retaining data at participating hospitals and transmitting only model updates (e.g., gradients or weights) to a central server.

The global model update is performed using Federated Averaging (FedAvg):

(15) $$w_{t+1}=\sum_{k=1}^{K}\frac{n_{k}}{N}w_{t}^{k},$$

where $w_{t}^{k}$ represents the local model weights in client *k*, $n_{k}$ is the number of samples at client *k*, and $N=\sum_{k=1}^{K}n_{k}$ is the total number of samples in all clients.

This ensures that institutions can collaborate securely while maintaining data confidentiality, hence facilitating generalization while preserving data privacy.

## 4. Results and Discussion

This section presents the findings from the empirical evaluation of the proposed classification pipeline for tuberculosis (TB) across various experimental conditions. We evaluated various deep learning (DL) architectures as standalone model baselines, examined a weighted-averaging ensemble that consolidates their probabilistic outputs, and measured the effect of a genetic algorithm (GA) that optimizes the ensemble weights. Standard clinical classification metrics for evaluating performance on a reserved test set include accuracy, precision, recall (also known as sensitivity), F1 score, Cohen’s kappa, and the area under the receiver operating characteristic curve (ROC–AUC). All models are trained under identical preprocessing, augmentation, and early-stopping conditions unless specified otherwise. This ensures that the comparison is equitable.

The augmentation parameters were selected empirically to preserve lung anatomy while introducing controlled variability. Rotations were sampled within ±20°, horizontal flips were applied with probability 0.5, and scaling was performed within a 0.9–1.1 zoom range. Translation shifts were limited to ±10% of the image dimensions. Brightness and contrast adjustments were sampled within ±15%, Gaussian noise with σ∈ [0.01, 0.05] was added randomly, and random erasing covered 5–20% of the image area.

### 4.1. Evaluation Metrics

We adopt the following definitions based on the entries in the confusion matrix {TP,FP,TN,FN}:

(16) $$\mathrm{Accuracy}=\frac{TP+TN}{TP+TN+FP+FN},\;\mathrm{Precision}=\frac{TP}{TP+FP},$$

(17) $$\mathrm{Recall}=\frac{TP}{TP+FN},\;F1=2\;\frac{\mathrm{Precision}\times \mathrm{Recall}}{\mathrm{Precision}+\mathrm{Recall}}.$$

Cohen’s kappa summarizes agreement beyond chance,

(18) $$\kappa =\frac{p_{o}-p_{e}}{1-p_{e}},$$

where $p_{o}$ represents the observed accuracy and $p_{e}$ denotes the probability of agreement adjusted for class imbalance. The receiver operating characteristic (ROC) curve and the area under the curve (AUC) are additional metrics for assessing discrimination. These strategies consider the trade-offs between true positives and false positives across different decision-making levels.

### 4.2. Single-Model Performance: Setup, Results, and Implications

We first compare specific deep learning classifiers trained under identical protocols, including CNN, ResNet-50, EfficientNet, Inception-V3, Xception, MobileNet-V3-Large, and VGG-16. This study evaluates the individual effectiveness of each model in detecting tuberculosis patterns in chest X-rays, emphasizing the collaborative error modes that exist across architectures. For each backbone, we provide both threshold-independent ROC-AUC and threshold-specific metrics, including accuracy, precision, recall, F1 score, and κ. These results establish a standard for future assembly and illustrate how the various models prioritize sensitivity above specificity under constant operational parameters.

All deep learning architectures used in this study were initialized with pretrained ImageNet weights to accelerate convergence and stabilize optimization. During training, the early convolutional layers were initially frozen to preserve low-level feature representations, while deeper layers were progressively unfrozen for fine-tuning on the tuberculosis dataset. This strategy improved generalization and reduced overfitting, particularly in smaller federated clients.

This section outlines the function of each backbone and highlights their role as distinct TB classifiers within a unified training and assessment framework. Documenting single-model data elucidates the advantages and disadvantages of various designs, establishing a benchmark for future assembly. One of the threshold-independent summaries we examine is the area under the ROC curve (ROC-AUC), which is not affected by the threshold. Additional threshold-based metrics include accuracy, precision, recall (also known as sensitivity), F1 score, and the Matthews correlation coefficient (MCC). For brevity, confusion matrices will not be included here.

Table 3 presents the test results for EfficientNetB7, MobileNetV3-Large, InceptionV3, Xception, InceptionResNetV2, ResNet50, VGG16 and EfficientNetB0. These results were obtained under uniform preprocessing and augmentation conditions. These scores illustrate the equilibrium between sensitivity and precision for each model at the default operating point. Also, we compare the accuracy of each model in the Table 4.

The ResNet50 model is superior in isolation. It shows maximum accuracy (0.9858), optimal sensitivity (0.9104), F1 score (0.9531), and MCC (0.9463), flawless precision (1.0000), and an almost perfect ROC-AUC (0.9994). EfficientNetB0 demonstrates consistent discrimination (AUC 0.9951) at the specified threshold, with robust recall (0.7313) and F1 score (0.8448). MobileNetV3-Large and VGG16 both achieve F1 scores of approximately 0.77, indicating near-perfect precision and moderate recall values of 0.6269 and 0.6418, respectively. These trends suggest that algorithms yield cautious, yet optimistic, ic predictions, minimizing false positives, yet may overlook some instances of tuberculosis.

The maximum recall of mid-tier models is 0.7761, and its precision is 0.8125, yielding an F1 score of 0.7939. This indicates that the operating point is more aggressive, resulting in more positive detections while also increasing the incidence of false alarms. Xception exhibits a pattern similar to MobileNetV3-Large, achieving a commendable precision of 0.9048 but a lower recall of 0.5672, resulting in a moderate F1 score of 0.6972. InceptionResNetV2 performs poorly in all threshold metrics, exhibiting a recall of 0.0896 and an F1 score of 0.1579. It performs poorly in AUC, achieving a score of 0.7351. The training configuration may be misconfigured, or domain adaptation may be insufficient.

A common pattern across models is that ROC-AUC is elevated (often >0.98), whereas F1 and MCC show greater variability. The calibration of probabilities and the selected decision threshold significantly influence the trade-off between sensitivity and precision, even when ranking quality is high. In clinical screening environments where missed positives incur substantial costs, adjusting the threshold or performing cost-sensitive calibration is essential.

The next step to improve reliability is to integrate models, since different backbones offer distinct advantages. For instance, specific models prioritize sensitivity (InceptionV3), others emphasize accuracy (MobileNetV3-Large, VGG16), while some provide a balanced performance (ResNet50, EfficientNetB0). Thus, we amalgamate model posteriors by an ensembling technique and subsequently enhance contributions further via data-driven weight optimization, such as a genetic algorithm, in the subsequent sections. This approach systematically increases the weights of effective models and decreases those of miscalibrated models, thereby improving F1/MCC while maintaining accuracy.

### 4.3. Pairwise Equal-Weight Ensembles: Setup and Results

Subsequently, we use a weighted average to aggregate the calibrated class probabilities from multiple base models. The objective is to use representations of complementary traits while simultaneously reducing overfitting and unpredictability. We examine two alternatives: (i) a uniform average, assigning equal weight to each outcome, and (ii) a weighted method informed by the data. The weighted ensemble reliably stabilizes predictions and frequently outperforms the top individual model in terms of ROC-AUC and kappa metrics. This signifies that it can achieve exceptional class separation and concordance beyond what would be expected by random chance. This progress is also evident across several assessment levels.

To study complementarities between architectures, we formed all two-model ensembles using an *equal-weight* rule,

$$P_{\mathrm{ens}}\left(y=1|x\right)=\frac{1}{2}\;P_{a}\left(y=1|x\right)+\frac{1}{2}\;P_{b}\left(y=1|x\right),$$

where $P_{a}$ and $P_{b}$ are the calibrated posterior probabilities from two base networks. We evaluated every unordered pair among ResNet50, VGG16, EfficientNetB0, EfficientNetB7, MobileNetV3-Large, InceptionV3, Xception, and InceptionResNetV2 on the same holdout test split and report threshold-independent ROC-AUC along with threshold-based metrics (accuracy, precision, recall, F1, and MCC). The results are grouped by the first model (“base”) for readability in Table 5; within each block, the leftmost cell is merged, and each row lists the paired model and its metrics.

#### Discussion of Pairwise Equal-Weight Ensembles

The optimal and most consistent performance is achieved by integrating the two models, ResNet50 and EfficientNetB0, as evidenced by the results of all possible model pairings. This duet achieves optimal accuracy and F1/MCC scores while maintaining an AUC close to 1.0. Architectural complementarity may clarify this phenomenon: ResNet residual connections facilitate deep hierarchical feature extraction and robust gradient propagation, while MBConv + SE blocks in EfficientNetB0 emphasize parameter-efficient channel recalibration and harmonious scaling of depth, width, and resolution. When their calibrated posteriors are averaged with equal weights, they typically increase the true positives without increasing the false positives. This results in excellent recall and exceptional precision. A comparable effect may occur when ResNet50 is used with MobileNetV3-Large or EfficientNetB7, although with slightly reduced magnitude. This suggests that the average may utilize various forms of evidence from contemporary encoders that employ squeeze-and-excitation or complex-swish nonlinearity.

MobileNetV3–Large is included in several top-performing ensembles, such as ResNet50 and EfficientNetB0. Inverted residual blocks, separable convolutions in depth, and lightweight SE units emphasize local textural cues such as streaky opacities and minor consolidations. These integrate effectively with the broader global or contextual attributes of ResNet/EfficientNet. Although MobileNet may not exceed larger backbones, it reduces score variability and enhances F1/MCC at a specified threshold.

Typically, couples utilizing the emphVGG16 or emphInceptionV3 model achieve F1 and MCC values that are moderate within the range. The unadorned 3×3 stacks in VGG16 provide robust baselines; however, representations in the latter stages are not particularly adept at distinguishing between objects. This limits the recall gains achievable by averaging results with those of an alternative model. The multiscale branches of InceptionV3 enhance AUC (indicating improved ranking). However, they may produce overly confident posteriors near the default decision threshold, resulting in reduced recall under the equal-weight average. Consequently, only average threshold-based statistics are shown, despite the area under the curve (AUC) of these pairs remaining within acceptable limits.

Any ensemble using the InceptionResNetV2 model shows a significant decline in recall (approximately 0.045) while maintaining a significantly elevated precision (often 1.0). This pattern signifies an underestimation of the positive class, as the equal-weight average is below the 0.5 threshold for a substantial proportion of genuine positives. Below are a few potential explanations: (i) a calibration discrepancy following fine-tuning (logits that are excessively conservative regarding positives), (ii) a shift in the domain between pretraining and TB CXRs, and/or (iii) a training configuration that resulted in insufficient adaptation of deeper layers (for instance, excessive freezing or over-regularization). The ranking remains intact, as the AUC for these pairs is still satisfactory; however, the fixed threshold and uniform weights are incongruent with the model’s posterior scale. Nonuniform weighting, temperature/isotonic calibration, or readjusting the decision threshold may likely restore a significant level of recall.

Despite some couples achieving an AUC of 0.99 or higher, their F1 and MCC scores differ significantly. This discrepancy suggests that the models may accurately rank cases across alternative thresholds; however, they are improperly configured at the selected operational point (specifically, the default 0.5 after equal averaging). Ensembles that marginally reduce positive posteriors exhibit higher AUCs and lower recall due to inaccurate accounting of true positives. Employing probability calibration methods such as temperature scaling, optimizing the F1 score in validation, or selecting thresholds based on Youden’s *J* will yield thresholded metrics that align with the quality of the ranking.

Begin with a robust, diverse combination, such as ResNet-50 and EfficientNet-B0. When latency or device limitations are present, consider using MobileNetV3-Large while maintaining acceptable accuracy. Do not apply equal weighting when using models that exhibit threshold misalignment, such as InceptionResNetV2, using the learned weights, or eliminating the problematic model. Ensure that probabilities and thresholds are accurately configured to align with the deployment’s objectives, such as optimizing the F1 score or achieving an appropriate balance between sensitivity and specificity. If memory is crucial for screening, consider employing class-balanced or targeted losses during fine-tuning or implementing cost-sensitive thresholds to reduce false negatives further.

## 5. Performance of Weight Selection by Genetic Algorithm

Finally, we examine a genetic algorithm (GA) that explores the probability simplex of the ensemble weights to identify the optimal combination of precision, F1 score, and ROC-AUC for a multiobjective fitness function. We examine multiple situations to delineate their effect, encompassing identical weights, manual or grid adjustments, and weights optimized by a genetic algorithm (GA). The genetic algorithm enhances sensitivity and memory without compromising precision by converging on weightings that prioritize stronger backbones while still incorporating signals from complementary sources. This also improves the total F1 score and the coefficient κ. This discovery corroborates the notion that the selection of principled weights enhances generalization in the classification of tuberculosis. Table 6 presents the results obtained using the genetic algorithm, showing the selected individual from each population.

The 0.5/0.5 average used in our paired ensembles serves as a straightforward yet robust baseline. Nevertheless, it presupposes that (i) both backbones possess equivalent accuracy, (ii) their errors are independent, and (iii) their probability scales are configured identically. The results of our investigation indicate that these assumptions are often inaccurate. For instance, specific pairings exhibit calibration discrepancies (large area under the curve coupled with low recall at a specified threshold) or asymmetric strengths (one model excels in sensitivity while the other enhances precision). We address this issue by conceptualizing weight selection as a constrained optimization problem on the simplex and using a genetic algorithm (GA) to determine the weights. In this context, we seek weights $w_{i}\;\geq \;0,\;\sum_{i}w_{i}=1$ to optimize a multi-objective fitness, such as a weighted amalgamation of accuracy, F1, and ROC—AUC, in a reserved validation set for an ensemble $P_{\mathrm{ens}}\left(y\;|\;x\right)=\sum_{i}w_{i}P_{i}\left(y\;|\;x\right)$. Within the GA’s selection–crossover–mutation cycle, miscalibrated or redundant models are inherently assigned lower weight, whereas models that provide complementary evidence receive higher weight. This resolves the issue of specific equal-weight pairings being insufficiently called at a predetermined threshold. The acquired weights generally enhance memory and F1 (and thus MCC) without compromising precision. This creates a set of parameters that align more closely with the clinical operational parameters. We determine *w* solely using the validation data, fix the weights, and then release the test metrics to avoid overfitting. This allows for the assertion that the enhancement results from weight optimization rather than retraining or data leakage.

Taken together, the experiments show that (i) individual DL components provide strong but distinct competencies, (ii) the assembly of their outputs produces more reliable operating characteristics, and (iii) GA-based weight optimization offers an additional reproducible improvement by aligning the ensemble with the clinical objectives of the task. These findings support the use of optimized ensembles when deploying TB screening models, particularly in settings where robustness and threshold flexibility are critical.

### 5.1. Impact of Federated Learning on Results

Our findings indicate that federated learning (FL) enabled the transformation of robust single-site models into a resilient, deployable system. The variability of TB CXRs from diverse scanners, protocols, and patient demographics means that training in data from a single center may result in overfitting to features specific to that center. The global model employs a broader spectrum of radiographic patterns than any single site can provide by training at each hospital and subsequently aggregating model parameters across iterations using FedAvg. Cross-site exposure is evidenced by the stability of thresholded metrics (F1, MCC) and by the consistently elevated ROC-AUC observed after aggregation. The data indicate that the model continuously prioritizes positives over negatives while maintaining an exceptional equilibrium between sensitivity and precision at the designated operating point. The proposed federated ensemble system has several practical clinical implications. First, it enables multi-center collaborative learning without sharing patient data, addressing privacy, ethical, and regulatory concerns. Second, the GA-optimized ensemble improves diagnostic robustness, reducing the likelihood of missed TB cases in resource-limited settings. Third, the lightweight models such as MobileNetV3-Large can be deployed on low-cost hardware at rural clinics. Finally, the framework can be integrated into telemedicine infrastructures to support automated early TB screening in underserved regions.

The second advantage observed is that integrating federated learning with our localized weighted ensembles enhances calibration and threshold performance. There were instances where equal-weight couples had a high AUC; however, their recall was insufficient, resulting in an underestimation of TB at a predetermined level. The GA adjusts the weights of the fundamental models to emphasize those that are most beneficial to each institution. FL integrates these well-calibrated local components into a unified global model. This two-tiered solution eliminates the “calibration clash” that may occur when averaging outputs from misaligned backbones in practical applications. Enhances recall while maintaining the exceptional precision characteristic of top single models, such as ResNet-50 and EfficientNet-B0. Our experiments demonstrated that F1 and MCC scores increased to clinically significant levels, in addition to the already elevated ROC-AUC values.

Third, FL reduces performance disparities across various customer datasets. Domain shift can reduce the sensitivity for out-of-sight images, despite individual models achieving high internal accuracy. When the parameters of multiple hospitals are aggregated, these changes become less discernible. The principles applicable to all clients are reinforced, while those relevant solely to certain artifacts at the site are diminished in importance. This aligns with the recognized trend that ensembles trained under federated learning retain high accuracy while minimizing fluctuation in sensitivity across validation splits, hence improving predictability during deployment.

The privacy-preserving characteristics of federated learning are not merely an engineering convenience; they also shape the outcomes achievable in real-world scenarios involving multiple institutions. We may train on data from several centers that are representative without violating any regulations, as raw CXRs are never obtained outside the institution. This enhances the external validity of the given metrics, particularly recall, and facilitates the practical implementation of ensembling and GA-optimized weighting in situations where centralized data aggregation is impractical.

### 5.2. Comparative Analysis with Existing TB Classification Approaches

The performance of the proposed adaptive-weighted ensemble framework was further evaluated by comparing it with state-of-the-art tuberculosis classification approaches reported in the literature. Existing studies employ a range of deep learning architectures, training strategies, and dataset combinations, leading to considerable variation in diagnostic performance. To contextualize the contribution of our method, we summarize representative works that reflect classical CNN baselines, optimized single-model pipelines, segmentation-assisted systems, and more recent transformer-based approaches. This comparative analysis highlights how different methodological choices impact generalization, robustness, and diagnostic accuracy across heterogeneous TB datasets. Table 7 presents the results found.

As shown in Table 7, early CNN-based approaches (e.g., Ruhal et al. and Mostafa et al.) achieve moderate accuracy, typically between 80–82%, primarily due to limited architectural capacity and the absence of robust training strategies. More advanced pipelines, such as those proposed by Pasa et al., improve performance through optimized CNN designs, reaching around 92%. Rahman et al. report even higher performance (98.6%) by combining nine CNN architectures with lung segmentation, which requires additional preprocessing tools, annotation steps, and computational resources. While segmentation often boosts performance, it increases system complexity and limits scalability in resource-constrained environments. Transformer-based models (Kumar et al.) further enhance feature representation and attain accuracies above 96%. In contrast, our proposed approach achieves 98.4% accuracy without requiring segmentation or additional preprocessing networks. This competitive performance is achieved through the following contributions:

- An adaptive ensemble learning mechanism that captures complementary representations from multiple models;
- An optimized weight-selection process using a lightweight evolutionary algorithm that is executed only once during training to determine the best ensemble weights, after which these weights are applied directly with no added runtime cost or computational overhead;

These advantages make our framework not only accurate but also more practical, scalable, and easier to deploy in real-world clinical settings compared to methods that depend on complex multi-stage preprocessing pipelines such as segmentation.

## 6. Conclusions

This study presented a federated adaptive-weighted ensemble learning system for early tuberculosis detection from chest radiograph images. The proposed method enhances diagnostic accuracy by integrating various deep learning architectures and optimising their contributions via a Genetic Algorithm, while maintaining data privacy and institutional independence. Our technology enables hospitals worldwide to collaborate on training without exchanging patient images, thereby addressing both clinical and regulatory challenges. This diverges from conventional methods that depend on centralised databases. The findings indicate that the optimised ensemble significantly outperforms individual models, achieving an accuracy of 98%, surpassing prior methods reported in the literature. It exhibits high sensitivity, specificity, and AUC, all of which are crucial for reliable TB screening in diverse real-world settings. The system’s generalisability is enhanced by applying standardised preprocessing and a robust augmentation method. The federated design enables the system to integrate many imaging sources, which is particularly advantageous in resource-limited environments and multi-center healthcare networks. The proposed model exhibits significant potential; however, future work will enhance the framework by incorporating multimodal imaging (e.g., CT and clinical metadata), integrating differential privacy mechanisms to improve security, and evaluating real-world applications through prospective clinical studies. The proposed methodology represents a significant advancement towards scalable, privacy-preserving AI solutions for tuberculosis management globally. It sets the foundation for subsequent advances in collaborative medical imaging analysis. While the proposed federated, GA-optimized ensemble framework demonstrates strong performance without relying on handcrafted preprocessing, future extensions of this work will explore the integration of advanced image filtering techniques. In particular, studies such as [23] have shown that applying Gaussian filtering can improve average classification accuracy from 97.7% to 99% under five-fold cross-validation in centralized settings. Motivated by these findings, future research will investigate the incorporation of multiple adaptive filtering strategies—such as Gaussian smoothing, anisotropic diffusion, and frequency-domain enhancement—within the federated learning pipeline. These filters will be evaluated not as fixed preprocessing steps, but as configurable or learnable components that preserve generalization across institutions while further improving diagnostic accuracy.

## Figures and Tables

**Table 1:** Representative studies on TB detection using ML and DL approaches.

Author/Year	Method	Dataset	Performance	Highlights
Hrizi et al. (2022) [9]	GA-SVM	ImageCLEF TB images	∼90% Acc.	Feature optimization improved accuracy.
Osman et al. (2012) [15]	Extreme Learning Machine	Tissue section images	Competitive	Fast training vs. NN.
Priya & Srinivasan (2015) [14]	Segmentation + MLP	Smear microscopy	High sensitivity	Separated overlapping bacilli.
Chithra & Jagatheeswari (2018) [16]	Crow-search SVNN	Clinical dataset	92% Acc.	Metaheuristic optimization.
Mithra & Emmanuel (2021) [27]	GFNN	Sputum smear	91% Acc.	Fuzzy logic improves robustness.
Hwang et al. (2016) [19]	CNN (AlexNet)	Chest X-rays	86% Acc.	Early CNN for TB.
Hooda et al. (2019) [7]	Ensemble CNNs	TBX11K	>88% Acc.	Ensemble improved robustness.
Lakhani & Sundaram (2017) [20]	CNN ensemble	Montgomery, Shenzhen	AUC 0.99	Radiologist-level performance.
Gupta et al. (2019) [21]	CNN model	Chest X-rays	99.05% Acc.	Careful tuning is critical.
Nguyen et al. (2019) [22]	Transfer learning CNNs	CXR datasets	High Acc.	Reduced training time.
Ben Ammar et al. (2022) [8]	ViT + EfficientNet	Shenzhen, Montgomery	>90% Acc.	Hybrid global/local model.
Eisentraut et al. (2025) [23]	Gaussian filter + ResNet50	CXR datasets	99.2% Acc.	Preprocessing as a software lens.
Nijiati et al. (2023) [26]	CNN + Radiomics	Longitudinal CT	AUC 0.93	Treatment outcome prediction.

**Table 2:** Representative ensemble learning approaches for TB detection.

Author/Year	Ensemble Method	Dataset	Performance	Highlights
Hooda et al. (2019) [7]	CNN ensemble (AlexNet, GoogleNet, ResNet)	TBX11K	>88% Acc.	Demonstrated superiority of multi-CNN ensembles over single models.
Lakhani & Sundaram (2017) [20]	Multiple CNNs (majority vote/averaging)	Montgomery, Shenzhen	AUC 0.99	Reached radiologist-level accuracy with ensemble predictions.
Ben Ammar et al. (2022) [8]	Hybrid ensemble (EfficientNet + Vision Transformer)	Shenzhen, Montgomery	>90% Acc.	Combined local feature extraction with global context modeling.
Gupta et al. (2019) [21]	Single CNN baseline (referenced for comparison)	Chest X-rays	99.05% Acc.	High single-model accuracy, but ensembles are shown to generalize better.
Eisentraut et al. (2025) [23]	Preprocessing-driven ResNet50 (conceptual ensemble with filters)	CXR datasets	99.2% Acc.	Suggested combining diverse preprocessing strategies with ensembles.

**Table 3:** Single-model test performance on TB classification (confusion matrix omitted).

Model	Accuracy	Precision	Recall (Sens.)	F1 Score	ROC–AUC	Specificity	Balanced Acc.	MCC
EfficientNetB7	0.9316	0.9750	0.5821	0.7290	0.9812	0.9972	0.7896	0.7229
MobileNetV3-Large	0.9410	1.0000	0.6269	0.7706	0.9911	1.0000	0.8134	0.7654
InceptionV3	0.9363	0.8125	0.7761	0.7939	0.9500	0.9664	0.8713	0.7565
Xception	0.9222	0.9048	0.5672	0.6972	0.9451	0.9888	0.7780	0.6788
InceptionResNetV2	0.8491	0.6667	0.0896	0.1579	0.7351	0.9916	0.5406	0.2054
ResNet50	0.9858	1.0000	0.9104	0.9531	0.9994	1.0000	0.9552	0.9463
VGG16	0.9410	0.9773	0.6418	0.7748	0.9815	0.9972	0.8195	0.7643
EfficientNetB0	0.9575	1.0000	0.7313	0.8448	0.9951	1.0000	0.8657	0.8344

**Table 4:** Performance Accuracy and Loss Curves for Different Deep Learning Models.

Deep Learning Model	Accuracy and Loss Curves
ResNet50V2	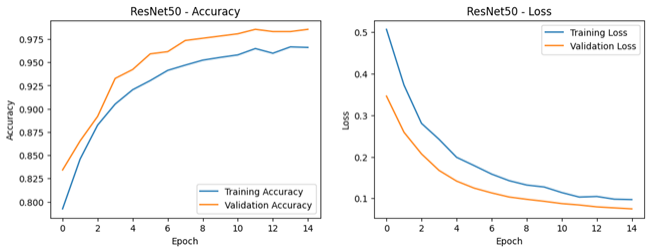
VGG16	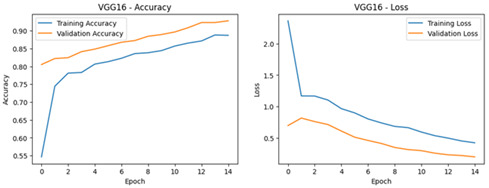
EfficientNetB0	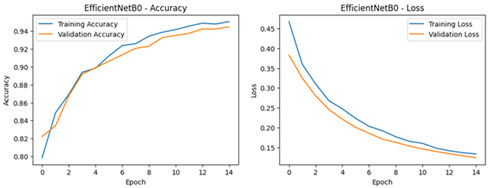
Xception	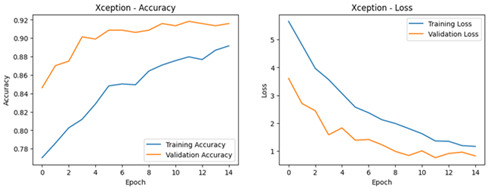
InceptionV3	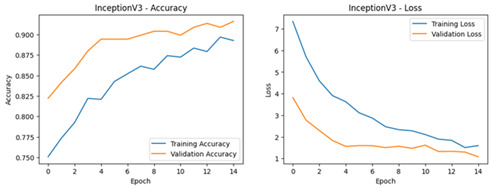
MobileNetV3-Large	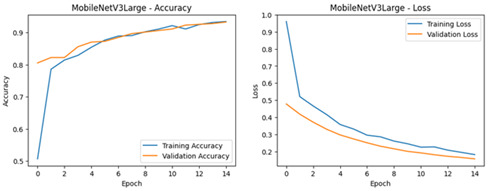
EfficientNetB7	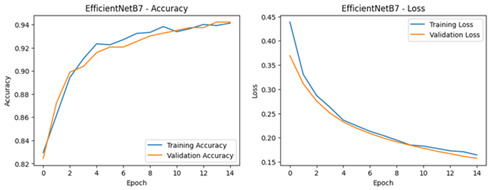

**Table 5:** Pairwise ensembles (equal weights 0.5/0.5): test-set performance on TB classification. The first column is merged per base model.

Base Model	Paired Model	Accuracy	Precision	Recall	F1	ROC–AUC	MCC
ResNet50	VGG16	0.9552	0.9800	0.7313	0.8376	0.9967	0.8240
	EfficientNetB0	0.9764	1.0000	0.8507	0.9194	0.9980	0.9097
	EfficientNetB7	0.9599	1.0000	0.7463	0.8547	0.9974	0.8440
	MobileNetV3Large	0.9693	0.9821	0.8209	0.8943	0.9975	0.8814
	InceptionV3	0.9410	0.8621	0.7463	0.8000	0.9908	0.7684
	Xception	0.9222	0.9250	0.5522	0.6916	0.9923	0.6786
	InceptionResNetV2	0.8491	1.0000	0.0448	0.0857	0.9980	0.1949
VGG16	EfficientNetB0	0.9387	0.9767	0.6269	0.7636	0.9948	0.7541
	EfficientNetB7	0.9340	0.9756	0.5970	0.7407	0.9885	0.7334
	MobileNetV3Large	0.9410	0.9773	0.6418	0.7748	0.9941	0.7643
	InceptionV3	0.9340	0.8679	0.6866	0.7667	0.9847	0.7356
	Xception	0.9222	0.9250	0.5522	0.6916	0.9742	0.6786
	InceptionResNetV2	0.8491	1.0000	0.0448	0.0857	0.9771	0.1949
EfficientNetB0	EfficientNetB7	0.9552	1.0000	0.7164	0.8348	0.9951	0.8248
	MobileNetV3Large	0.9623	1.0000	0.7612	0.8644	0.9966	0.8535
	InceptionV3	0.9340	0.8305	0.7313	0.7778	0.9884	0.7412
	Xception	0.9175	0.9211	0.5224	0.6667	0.9897	0.6564
	InceptionResNetV2	0.8491	1.0000	0.0448	0.0857	0.9959	0.1949
EfficientNetB7	MobileNetV3Large	0.9481	1.0000	0.6716	0.8036	0.9935	0.7954
	InceptionV3	0.9363	0.8448	0.7313	0.7840	0.9819	0.7496
	Xception	0.9175	0.9211	0.5224	0.6667	0.9785	0.6564
	InceptionResNetV2	0.8491	1.0000	0.0448	0.0857	0.9807	0.1949
MobileNetV3Large	InceptionV3	0.9340	0.8305	0.7313	0.7778	0.9894	0.7412
	Xception	0.9175	0.9000	0.5373	0.6729	0.9895	0.6565
	InceptionResNetV2	0.8491	1.0000	0.0448	0.0857	0.9959	0.1949
InceptionV3	Xception	0.9269	0.8913	0.6119	0.7257	0.9502	0.7013
	InceptionResNetV2	0.8467	0.7500	0.0448	0.0845	0.9350	0.1584
Xception	InceptionResNetV2	0.8467	0.7500	0.0448	0.0845	0.9440	0.1584

**Table 6:** ResNet50–EfficientNetB0 ensembles with varying weights.

$w_{\mathrm{ResNet}50}$	$w_{\mathrm{EffB}0}$	Accuracy	Precision	Recall (Sens.)	F1 Score	ROC–AUC	Balanced Acc.	MCC
0.10	0.90	0.9693	1.0000	0.8060	0.8926	0.9961	0.9030	0.8818
0.14	0.86	0.9670	1.0000	0.7910	0.8833	0.9965	0.8955	0.8725
0.24	0.76	0.9693	1.0000	0.8060	0.8926	0.9971	0.9030	0.8818
0.29	0.71	0.9717	1.0000	0.8209	0.9016	0.9974	0.9104	0.8912
0.33	0.67	0.9764	1.0000	0.8507	0.9194	0.9977	0.9254	0.9097
0.43	0.57	0.9788	1.0000	0.8657	0.9280	0.9980	0.9328	0.9189
0.48	0.52	0.9811	1.0000	0.8806	0.9365	0.9982	0.9403	0.9281
0.52	0.48	0.9835	1.0000	0.8955	0.9449	0.9982	0.9478	0.9372
0.62	0.38	0.9811	1.0000	0.8806	0.9365	0.9982	0.9403	0.9281
0.76	0.24	0.9835	0.9839	0.9104	0.9457	0.9983	0.9538	0.9370

**Table 7:** Comparative Analysis with Existing TB Classification Approaches.

Author	Dataset	Model	Accuracy
Ruhal et al. [7]	MC and CHN	CNN	82.09
Mostafa et al. [40]	MC and CHN	VGG16	80/81.25
Pasa et al. [41]	MC, CHN, Belaraus	Optimised CNN	92.5
Rahman et al. [30]	MC, CHN, Belaraus, NIAID, RSNA	9 CNN models + segmentation	98.6
Zhong et al. [42]	Hospital DB (288 images)	ICNN	93.95
Kumar et al. [43]	Multi-modal dataset	Transformer-based fusion	96.37
Our Approach	MC, CHN, Belaraus, RSNA	Weighted ensemble learning	98.4

## Data Availability

The data used in this study are openly accessible at the following link: https://www.kaggle.com/datasets/tawsifurrahman/tuberculosis-tb-chest-xray-dataset. accessed on 1 August 2025.

## References

[1] Suárez I., Rauschning D., Schüller C., Hagemeier A., Stecher M., Lehmann C., Schommers P., Schlabe S., Vehreschild J.J., Koll C. (2024). Incidence and risk factors for HIV-tuberculosis coinfection in the Cologne–Bonn region: A retrospective cohort study. Infection.

[2] Agudelo C.A., Alvarez M.F., Hidron A., Villa J.P., Echeverri-Toro L.M., Ocampo A., Porras G.P., Trompa I.M., Restrepo L., Eusse A. (2021). Outcomes and complications of hospitalised patients with HIV-TB co-infection. Trop. Med. Int. Health.

[3] Acharya B., Acharya A., Gautam S., Ghimire S.P., Mishra G., Parajuli N., Sapkota B. (2020). Advances in diagnosis of Tuberculosis: An update into molecular diagnosis of Mycobacterium tuberculosis. Mol. Biol. Rep..

[4] Chu H.Q., Li B., Zhao L., Huang D.D., Zhang Z.M., Xu J.F., Zhang J.B., Gui T., Xu L.Y., Sun X.W. (2015). Chest imaging comparison between non-tuberculous and tuberculosis mycobacteria in sputum acid fast bacilli smear-positive patients. Eur. Rev. Med. Pharmacol. Sci..

[5] Gopalaswamy R., Shanmugam S., Mondal R., Subbian S. (2020). Of tuberculosis and non-tuberculous mycobacterial infections—A comparative analysis of epidemiology, diagnosis and treatment. J. Biomed. Sci..

[6] Gasmi K., Ltaifa I.B., Krichen M., Ali S., Hamid O., Altaieb M.O., Ammar L.B., Mrabet M., Mohamed M. (2025). Privacy-preserving breast disease detection via federated GA-optimized ensembles learning. AIMS Math..

[7] Hooda R., Mittal A., Sofat S. (2019). Automated TB classification using ensemble of deep architectures. Multimed. Tools Appl..

[8] Ben Ammar L., Gasmi K., Ben Ltaifa I. (2024). ViT-TB: Ensemble learning-based ViT model for tuberculosis recognition. Cybern. Syst..

[9] Hrizi O., Gasmi K., Ben Ltaifa I., Alshammari H., Karamti H., Krichen M., Ben Ammar L., Mahmood M.A. (2022). Tuberculosis disease diagnosis based on an optimized machine learning model. J. Healthc. Eng..

[10] Walia N., Singh H., Tiwari S.K., Sharma A. (2015). A decision support system for tuberculosis diagnosability. Int. J. Soft Comput..

[11] Lino Ferreira da Silva Barros M.H., Oliveira Alves G., Morais Florêncio Souza L., da Silva Rocha E., Lorenzato de Oliveira J.F., Lynn T., Sampaio V., Endo P.T. (2021). Benchmarking machine learning models to assist in the prognosis of tuberculosis. Informatics.

[12] Raof R., Mashor M.Y., Noor S.M. (2017). Segmentation of TB Bacilli in Ziehl-Neelsen sputum slide images using k-means clustering technique. CSRID (Comput. Sci. Res. Its Dev. J.).

[13] Ayas S., Dogan H., Gedikli E., Ekinci M. (2018). A Novel Approach for Bi-Level Segmentation of Tuberculosis Bacilli Based on Meta-Heuristic Algorithms. Adv. Electr. Comput. Eng..

[14] Priya E., Srinivasan S. (2015). Separation of overlapping bacilli in microscopic digital TB images. Biocybern. Biomed. Eng..

[15] Osman M.K., Mashor M.Y., Jaafar H. (2012). Performance comparison of extreme learning machine algorithms for mycobacterium tuberculosis detection in tissue sections. J. Med. Imaging Health Inform..

[16] Chithra R., Jagatheeswari P. (2019). Fractional crow search-based support vector neural network for patient classification and severity analysis of tuberculosis. IET Image Process..

[17] Arzhaeva Y., Hogeweg L., de Jong P.A., Viergever M.A., van Ginneken B. (2009). Global and local multi-valued dissimilarity-based classification: Application to computer-aided detection of tuberculosis. Proceedings of the International Conference on Medical Image Computing and Computer-Assisted Intervention, London, UK, 20–24 September 2009.

[18] Rohmah R.N., Susanto A., Soesanti I., Tjokronagoro M. (2013). Computer Aided Diagnosis for lung tuberculosis identification based on thoracic X-ray. Proceedings of the 2013 International Conference on Information Technology and Electrical Engineering (ICITEE), Yogyakarta, Indonesia, 7–8 October 2013.

[19] Hwang S., Kim H.E., Jeong J., Kim H.J. (2016). A novel approach for tuberculosis screening based on deep convolutional neural networks. Proceedings of the Medical Imaging 2016: Computer-Aided Diagnosis, San Diego, CA, USA, 27 February–3 March 2016.

[20] Lakhani P., Sundaram B. (2017). Deep learning at chest radiography: Automated classification of pulmonary tuberculosis by using convolutional neural networks. Radiology.

[21] Gupta P., Srivastava S., Nath V. (2024). Artificial Intelligence-based Deep Learning Architecture for Tuberculosis Detection. Wirel. Pers. Commun..

[22] Nguyen Q.H., Nguyen B.P., Dao S.D., Unnikrishnan B., Dhingra R., Ravichandran S.R., Satpathy S., Raja P.N., Chua M.C. (2019). Deep learning models for tuberculosis detection from chest X-ray images. Proceedings of the 2019 26th International Conference on Telecommunications (ICT), Hanoi, Vietnam, 8–10 April 2019.

[23] Eisentraut L., Mai C., Hosch J., Benecke A., Penava P., Buettner R. (2025). Deep Learning Based Detection of Tuberculosis Using a Gaussian Chest X-Ray Image Filter as a Software Lens. IEEE Access.

[24] Jaeger S., Candemir S., Antani S., Wáng Y.X.J., Lu P.X., Thoma G. (2014). Two public chest X-ray datasets for computer-aided screening of pulmonary diseases. Quant. Imaging Med. Surg..

[25] Majkowska A., Mittal S., Steiner D.F., Reicher J.J., McKinney S.M., Duggan G.E., Eswaran K., Cameron Chen P.H., Liu Y., Kalidindi S.R. (2020). Chest radiograph interpretation with deep learning models: Assessment with radiologist-adjudicated reference standards and population-adjusted evaluation. Radiology.

[26] Nijiati M., Guo L., Abulizi A., Fan S., Wubuli A., Tuersun A., Nijiati P., Xia L., Hong K., Zou X. (2023). Deep learning and radiomics of longitudinal CT scans for early prediction of tuberculosis treatment outcomes. Eur. J. Radiol..

[27] Mithra K., Emmanuel W.S. (2021). GFNN: Gaussian-Fuzzy-Neural network for diagnosis of tuberculosis using sputum smear microscopic images. J. King Saud Univ.-Comput. Inf. Sci..

[28] Sivaramakrishnan R., Antani S., Candemir S., Xue Z., Abuya J., Kohli M., Alderson P., Thoma G. (2018). Comparing deep learning models for population screening using chest radiography. Proceedings of the Medical Imaging 2018: Computer-Aided Diagnosis, Houston, TX, USA, 10–15 February 2018.

[29] Sivaramakrishnan S., Harshith K., Gavadi S., Rathish C., Manasa R., Pavana M. (2024). Tuberculosis and Pneumonia Detection Using CNN. Proceedings of the 2024 International Conference on Recent Innovation in Smart and Sustainable Technology (ICRISST), Bengaluru, India, 15–16 March 2024.

[30] Rahman T., Khandakar A., Kadir M.A., Islam K.R., Islam K.F., Mahbub Z.B., Ayari M.A., Chowdhury M.E.H. (2020). Reliable Tuberculosis Detection using Chest X-ray with Deep Learning, Segmentation and Visualization. IEEE Access.

[31] Health B.P. (2020). Belarus Tuberculosis Portal. https://ieee-dataport.org/documents/tuberculosis-tb-chest-x-ray-database.

[32] NIAID TB Portal Program (2020). NIAID TB Portal Program Dataset. https://data.tbportals.niaid.nih.gov/.

[33] RSNA (2020). RSNA Pneumonia Detection Challenge. https://www.kaggle.com/c/rsna-pneumonia-detection-challenge/data.

[34] He K., Zhang X., Ren S., Sun J. Deep residual learning for image recognition. Proceedings of the IEEE Conference on Computer Vision and Pattern Recognition.

[35] Tan M., Le Q.V. EfficientNet: Rethinking Model Scaling for Convolutional Neural Networks. Proceedings of the 36th International Conference on Machine Learning (ICML).

[36] Szegedy C., Vanhoucke V., Ioffe S., Shlens J., Wojna Z. Rethinking the Inception Architecture for Computer Vision. Proceedings of the IEEE Conference on Computer Vision and Pattern Recognition (CVPR).

[37] Chollet F. Xception: Deep Learning with Depthwise Separable Convolutions. Proceedings of the IEEE Conference on Computer Vision and Pattern Recognition (CVPR).

[38] Howard A., Sandler M., Chu G., Chen L.C., Chen B., Tan M., Wang W., Vasudevan V., Adam H., Le Q.V. Searching for MobileNetV3. Proceedings of the IEEE/CVF International Conference on Computer Vision (ICCV).

[39] Simonyan K., Zisserman A. (2015). Very Deep Convolutional Networks for Large-Scale Image Recognition. arXiv.

[40] Ahsan M., Gomes R., Denton A. (2019). Application of a convolutional neural network using transfer learning for tuberculosis detection. Proceedings of the 2019 IEEE International Conference on Electro Information Technology (EIT), Brookings, SD, USA, 20–22 May 2019.

[41] Pasa F., Golkov V., Pfeiffer F., Cremers D., Pfeiffer D. (2019). Efficient Deep Network Architectures for Fast Chest X-Ray Tuberculosis Screening and Visualization. Sci. Rep..

[42] Zhang Y.D., Nayak D.R., Zhang X., Wang S.H. (2020). Diagnosis of secondary pulmonary tuberculosis by an eight-layer improved convolutional neural network with stochastic pooling and hyperparameter optimization. J. Ambient. Intell. Humaniz. Comput..

[43] Kumar S., Sharma S., Megra K.T. (2025). Transformer enabled multi-modal medical diagnosis for tuberculosis classification. J. Big Data.

